# High Genotypic Discordance of Concurrent *Mycobacterium tuberculosis* Isolates from Sputum and Blood of HIV-Infected Individuals

**DOI:** 10.1371/journal.pone.0132581

**Published:** 2015-07-15

**Authors:** Willy Ssengooba, Frank G. Cobelens, Lydia Nakiyingi, Gerald Mboowa, Derek T. Armstrong, Yukari C. Manabe, Moses L. Joloba, Bouke C. de Jong

**Affiliations:** 1 Department of Medical Microbiology, College of Health Sciences Makerere University, Kampala, Uganda; 2 Department of Global Health and Amsterdam Institute of Global Health and Development, Academic Medical Center, University of Amsterdam, Amsterdam, Netherlands; 3 Unit of Mycobacteriology Institute of Tropical Medicine, Antwerp, Belgium; 4 KNCV Tuberculosis Foundation, The Hague, Netherlands; 5 Infectious Diseases Institute, College of Health Sciences Makerere University, Kampala, Uganda; 6 Johns Hopkins University School of Medicine, Baltimore, MD, United States of America; 7 Division of Infectious Diseases, New York University, New York, NY, United States of America; National Institute of Infectious Diseases, JAPAN

## Abstract

**Background:**

Among HIV-infected individuals with CD4 less than 200 cells/mm^3^, tuberculosis often has an atypical presentation, is more likely to be disseminated and is diagnostically challenging. We sought to understand the genotypic discordance of concurrent sputum and blood *M*. *tuberculosis* (MTB) isolates from HIV-infected individuals.

**Methods:**

From a prospective diagnostic accuracy study with 182 HIV-infected culture-positive TB adults, isolates were obtained from 51 of 66 participants who were MTB culture-positive by both sputum and blood. Isolates were subjected to susceptibility testing to 1^st^ line drugs, spoligotyping and 24 locus- MIRU-VNTR.

**Results:**

The median age of the participants was 31 (IQR; 27–38) years and 51% were male. The median CD4 count was 29 (IQR; 10–84) cells/mm^3 ^ with 20% taking ART; 8.0% were previously treated for TB, and 63% were AFB smear-negative. The isolates belonged to two of the main global MTB-lineages; East-African-Indian (L3) 17 (16.7%) and Euro-American (L4) 85 (83.3%). We identified 26 (51.0%) participants with discordant MTB-genotypes between sputum and blood, including two patients with evidence of mixed infection in either compartment. Having discordant MTB-genotypes was not predicted by the MTB-lineage in either blood or sputum, CD4 cell count, or any other clinical characteristic.

**Conclusions:**

There is a high genotypic discordance among *M*. *tuberculosis* concurrently isolated from sputum and blood of HIV-infected individuals. These findings suggest that infection with more than one strain of *M*. *tuberculosis* occurs in at least half of patients with advanced HIV infection.

## Background

The ongoing tuberculosis pandemic is in part sustained by the presence of a large population of individuals with impaired immunity as a result of AIDS [1]. Though antibiotic therapy is effective in these patients when infected with drug-sensitive strains, peculiarities in the course of infection present challenges for both diagnosis and therapy [2–4]. Specifically, the immune impaired patient is less likely to show sputum smear positivity and more likely to present with advanced and/or disseminated disease [5, 6]. For these reasons, there is great interest in better understanding the course of tuberculosis in the immune impaired to improve diagnosis and therapy.[7, 8].

Patients with both mycobacteremia and pulmonary involvement have increased mortality rates [2]. One aspect of the greater propensity for disseminated tuberculosis in the immune impaired is the potential for polyclonal infection, as different strains of *M*. *tuberculosis* may have distinct fitness in different host niches [9–12]. Infections with more than one genotype challenge old dogmas related to TB immunity, pathogenesis and progression from latent to active TB. They moreover raise questions on the timing of multiple infections and the mechanism of reactivation of both infections simultaneously, which we expected to be higher in patients in whom the immune system is impaired. Such different genotypes in different samples may be clinically relevant. Animal studies show that different strains differ in the severity of disease they cause [13–15], and in humans a subset of the Euro-American MTB lineage was found to be less common in TB meningitis than in pulmonary TB, suggesting an interaction between bacterial genotype and clinical phenotype[16, 17]. Moreover, multidrug resistant and extensively drug resistant (MDR/XDR) strains have been isolated from blood of HIV-infected individuals with low CD4 cell counts [8], and mixed infections of susceptible and MDR strains of different genotype isolated from either sputum or blood have been reported[18]. Strains involved in mixed infections may consequently have different susceptibility patterns, requiring treatment to be adjusted to the most resistant strain that can however be missed if only one body compartment is sampled [19, 20]. We thus investigated what proportion of patients with poor cellular immunity were infected with different MTB strains in sputum as compared to blood.

## Materials and Methods

### Study Population

Clinical and laboratory culture data were obtained from a prospective cohort of HIV-infected outpatients at the Infectious Diseases Institute [IDI] and HIV-infected inpatients from Mulago National Tertiary Referral Hospital, both in Kampala, Uganda, in whom the diagnosis of pulmonary and extrapulmonary tuberculosis (EPTB) was considered [21]. Of the 506 participants recruited, 69% (351/506) were inpatients. Participants were 18 years and older and provided two spot sputum samples and a blood sample for mycobacterial culture and CD4 cell count at the baseline visit. The study had 104 individuals with positive sputum MTB culture only, 66 with concurrently positive sputum and blood cultures and 12 with positive blood culture only. No individual was found to have non-tuberculous mycobacteria in blood culture.

For the present study, we only considered the 66 TB patients with both sputum and blood culture positive results for *M*. *tuberculosis*.

### Laboratory Procedures

Mycobacterial cultures of sputum and blood were done at the Department of Medical Microbiology, Makerere University, Uganda, according to standard procedures as described elsewhere [21].

All Capilia TB Neo (TAUN, Numazu, Japan) and/or Ziehl-Neelsen (ZN) positive samples were confirmed as *M*. *tuberculosis complex* (MTBc) by PCR detection of a 500 bp fragment of the IS6110[22] and then the isolates were subjected to drug susceptibility testing (DST) to streptomycin, isoniazid, rifampicin and ethambutol using the proportion method on solid media. DST was done partly at the Department of Medical Microbiology, Makerere University, and at the Institute of Tropical Medicine (ITM), Antwerp, Belgium. The isolates resistant to rifampicin and/or isoniazid from the batch tested from Uganda were retested at ITM and repeat results were considered final.

#### *M*. *tuberculosis* strain typing methods

To determine *M*. *tuberculosis* strain types from sputum and blood spoligotyping, using boiled bacterial lysates, was performed at the Mycobacteriology Unit at ITM according to standard procedures [23]. Spoligotyping (spacer oligonucleotide typing) is a PCR-based method that can be simultaneously used for detection as well as typing of the MTBc basing on the amplification of a highly polymorphic Direct Repeat (DR) locus in *M*. *tuberculosis* genome. Although it is well suited for discrimination of clinical isolates the method has clear disadvantages for the investigation of the deep phylogenetic structure. For a finer phylogenetic classification, Variable Number of Tandem Repeats (VNTR)-typing applying genetic elements called Mycobacterial Interspersed Repetitive Units (MIRU) as genetic markers was performed on bacterial lysates at Genoscreen (Lille, France) [24]. MIRU-VNTR typing can provide unique high-resolution insights into the population structure of the MTBC, provides clear criteria for the identification of the different MTBc lineages and sub-lineages, and is best suited to identify instances of mixed infection in the same culture. The combination of both methods provides a higher resolution to identify clonal infection, akin to IS6110 RFLP[23, 25]. We performed both spoligotyping and MIRU-VNTR aiming at around 99% specificity and 95% sensitivity, to rigorously discriminate clonal variants of the paired samples, as previously documented[26].

#### Quality assurance and quality control

Samples were collected according to internationally accepted standards from the Uganda site only in a good clinical- and good laboratory practice compliant multicentre clinical trial registered under clinical trials.gov number NCT01525134 [21]. Blood culture samples were collected directly into Myco F/Lytic media, (Becton and Dickson, Franklin Lakes, NJ USA) culture bottles and incubated without further manipulation.

To rule-out cross-contamination, blinded samples of artificial sputum (mock sterile samples made of eggs and methylcellulose, Sigma-Aldrich MO267) were introduced in each batch of 10 sputum samples before processing and processed as other patient samples. There was no growth on any artificial sputa processed during the study period. For spoligotype analysis, samples were received in two batches, and eight of the samples from the first batch were blindly included in the second batch and re-analyzed blindly. All eight samples had identical spoligotype results on repeat. Also to rule out contamination from other activities in the laboratory during spoligotyping analysis, DNA from BCG and of H37Rv were included in each batch and none of the patient samples showed spoligotypes characteristic to either of the control strains.

### Data Analysis

Spoligotyping data were entered in a binary code format and the 24 loci Mycobacterial Interspersed Repetitive Unit-Variable-Number Tandem Repeat (MIRU-VNTR) data into Microsoft excel. The data were then imported to “MIRU-VNTR plus” online database (http://www.miru-vntrplus.org/MIRU)[27, 28] for lineage assignment. The MIRU database unassigned lineages were interpreted using another online database [29]. For spoligotyping data, we considered pairs with more than one spacer difference to be discordant MTB-genotypes. For 24 locus MIRU-VNTR, we considered data with interpretable results for at least 15 of the 24 loci; isolate pairs with more than one locus difference were considered discordant MTB-genotypes. For final MTB-lineage assignment, we considered results of spoligotype and/or MIRU-VNTR. For patients with both MIRU-VNTR and spoligotyping results, we considered MIRU-VNTR results for final interpretation of concordance. Mixed infection was defined as isolates with MIRU patterns with more than one allele per locus present in more than one locus per patient, otherwise MIRU-VNTR profiles with double alleles at a single locus were considered to be clonal variants of the same strain. Genotyping results were added to participant’s demographic data in Microsoft excel and data cleaned. Discrepancies were solved by checking the entries against the raw data. To visualize the phylogenetic distribution of MTB lineages/sub lineages, an unweighted pair-group method with arithmetic mean (UPGMA) tree was generated [27]. Data were exported to Epi Info 7.1.4 for analysis of frequencies and proportions of phenotypic and genotypic diversity of MTB from sputum and/or blood in relation to individual clinical characteristics. We compared patients with discordant sputum/blood genotype pairs with those of concordant pairs for differences in lineage, site of disease and patient characteristics caclulating odds ratios and using the 2-sided Fisher’s exact test. We attempted multivariate logistic regression to control for confounding but numbers were too small to build mathematically stable models. A *p*-value <0.05 was considered significant.

### Ethics Statement

This was a nested study within a study that was approved by the Joint Clinical Research Center Institutional Review Board (IRB) and the Uganda National Council of Science and Technology (UNCST, HS850). Each participant gave a written informed consent for participation in the main study, including a second written informed consent for the samples and isolates to be used in future studies. Additional IRB approval was obtained from the Institute of Tropical Medicine (ITM, 938/14), Antwerp, Belgium for the present genotyping analyses.

## Results

### Characteristics of study participants

**Fig 1:**
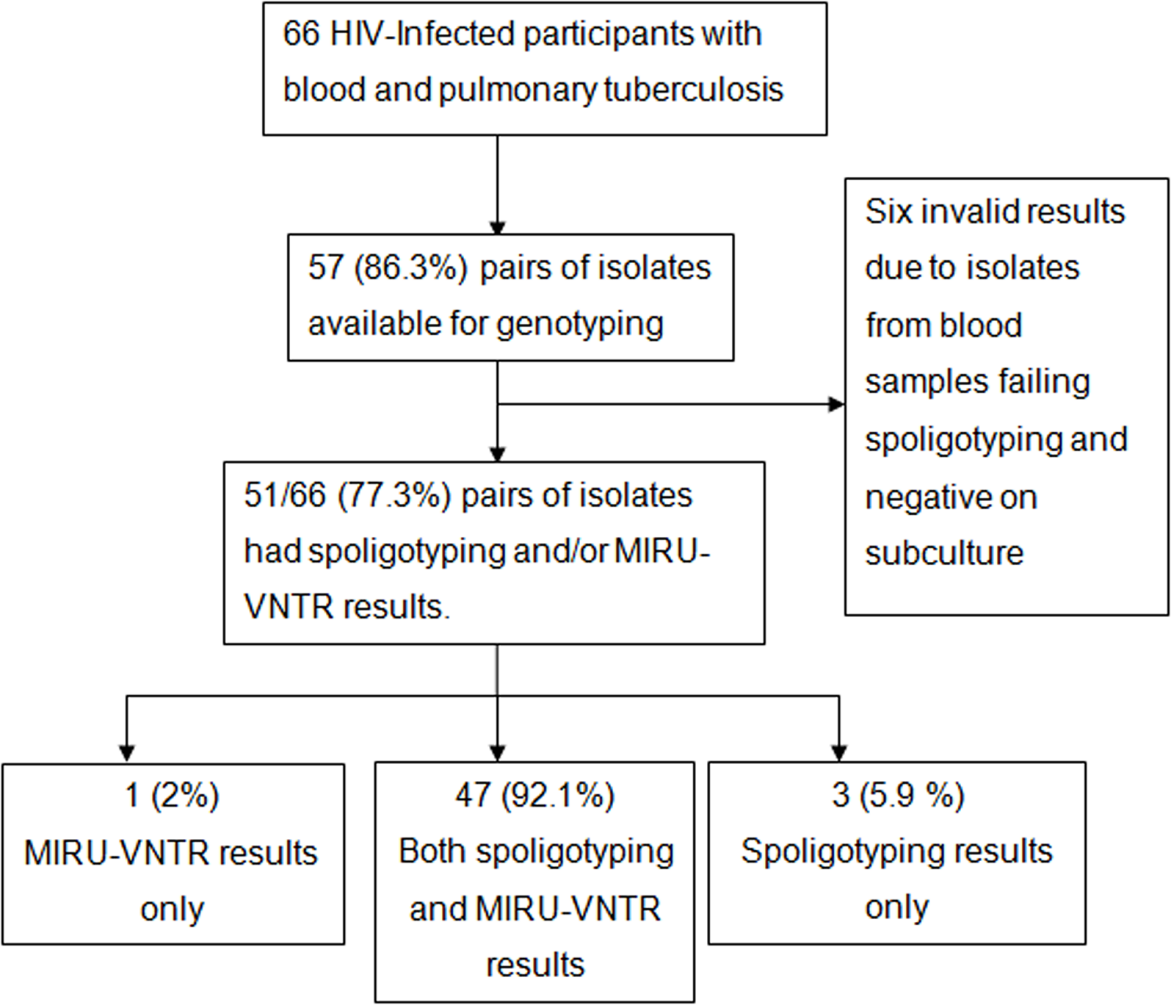
Flow chart showing the HIV-infected participants with concurrent sputum and blood *Mycobacterium tuberculosis*. Abbreviation; MIRU-VNTR= Mycobacterial Interspersed Repetitive Unit-Variable-Number Tandem Repeat.

Of the 66 HIV-infected participants’ in the parent study with concurrently positive sputum and blood cultures, 57 (86.3%) pairs of frozen isolates (from sputum and blood) were available, of which 51 pairs had sufficient viable bacilli/DNA available for interpretable pairwise spoligotyping and/or MIRU VNTR results, Fig 1).

Of these 51 participants, the median age was 31 years (interquartile range, IQR; 27-38) and 26 (51.0%) were male. The median CD4 cell count/mm^3^ was 29 (IQR; 10-84), the majority (33, 64.7%) had CD4 cell count <50 cells/mm^3^, and 31 (63.3%) were smear-negative by direct fluorescent smear microscopy. The median time to culture positivity in mycobacterial growth indicator tube (MGIT) on sputum was 10 (IQR; 6-12) days versus 24 (IQR; 21-30) days for blood cultures, and the majority of sputum samples 29 (56.9%) had 10-100 Lowenstein Jensen (LJ) culture colony counts, Table 1.

**Table 1:** Characteristic of participants with *M*. *tuberculosis* concurrently isolated from sputum and blood samples (n=51).

Parameter	Category	Frequency	Percent
**Age (years)**	<30	19	37.3
	30-39	22	43.4
	=/>40	10	19.6
	Median (IQR)	31 (27-38)	
**Sex**	Male	26	51.0
**CD4 cell count/mm** ^**3**^	<50	33	64.7
	51-200	15	29.4
	>200	3	5.9
	Median (IQR)	29 (10-84)	
**On ART (n=50)**	At enrollment	10	19.61
**TB treatment**	Previously treated	4	7.84
**Karnofsky performance score at baseline**	50-70	45	88.2
	80 and above	6	11.8
	Median (IQR)	60 (60-70)	
**Weight at baseline in Kgs**	Median (IQR)	50 (45-54.3)	
**Direct fluorescent smear microscopy (n=49)**	Negative	31	63.3
	Positive	18	36.7
**LJ (sputum) culture colony counts**	Negative	9	17.6
	<10	10	19.6
	10-100	29	56.9
	>100	3	5.9
**MGIT (sputum) culture time to detection (days)**	=/>12	15	29.4
	7-11	19	37.3
	</= 6	17	33.3
	Median (IQR)	10 (6-12)	
**Blood culture time to detection (days)**	=/> 30	14	27.5
	22-29	23	45.1
	</= 21	14	27.5
	Median (IQR)	24 (21-30)	

Abbreviations: IQR = inter quartile range, LJ- Lowenstein Jensen, MGIT- Mycobacterial Growth Indicator Tube.

### Distribution of *M*. *tuberculosis* lineages of concurrent isolates from sputum and blood samples

Of the 102 isolates from 51 TB patients, 17 (16.7%) belonged to the East-African-Indian lineage (Lineage 3, L3) and 85 (83.3%) to the Euro-American lineage (Lineage 4, L4). Among the L4 strains the most common sub-lineage was T2 (49; 57.6%). Among the L3 strains the most common sub-lineages were CAS and CAS1_DELHI both at 7 (41.2%). There were no significant associations between lineage or sub-lineage with site of disease, Table 2.

**Table 2:** Distribution of the *M*. *tuberculosis* lineages/sub-lineages concurrently isolated from sputum and blood samples.

Global MTB Lineage	Sub-lineage	Blood (N=51)	Sputum (N=51)	P-value*
**East-African-Indian lineage**	CAS	10(19.6)	7 (13.7)	0.425
**Euro-American lineage**	LAM	6 (11.8)	8 (15.7)	0.646
	T	31(60.8)	33 (64.7)	0.681
	Others†	4 (7.8)	3 (5.9)	0.695

Abbreviations; CAS= Central- Asian strain, LAM= Latin American and Mediterranean.

† = Haarlem, X2 and U sub-lineages

*2-sided Fisher’s exact

**Fig 2:**
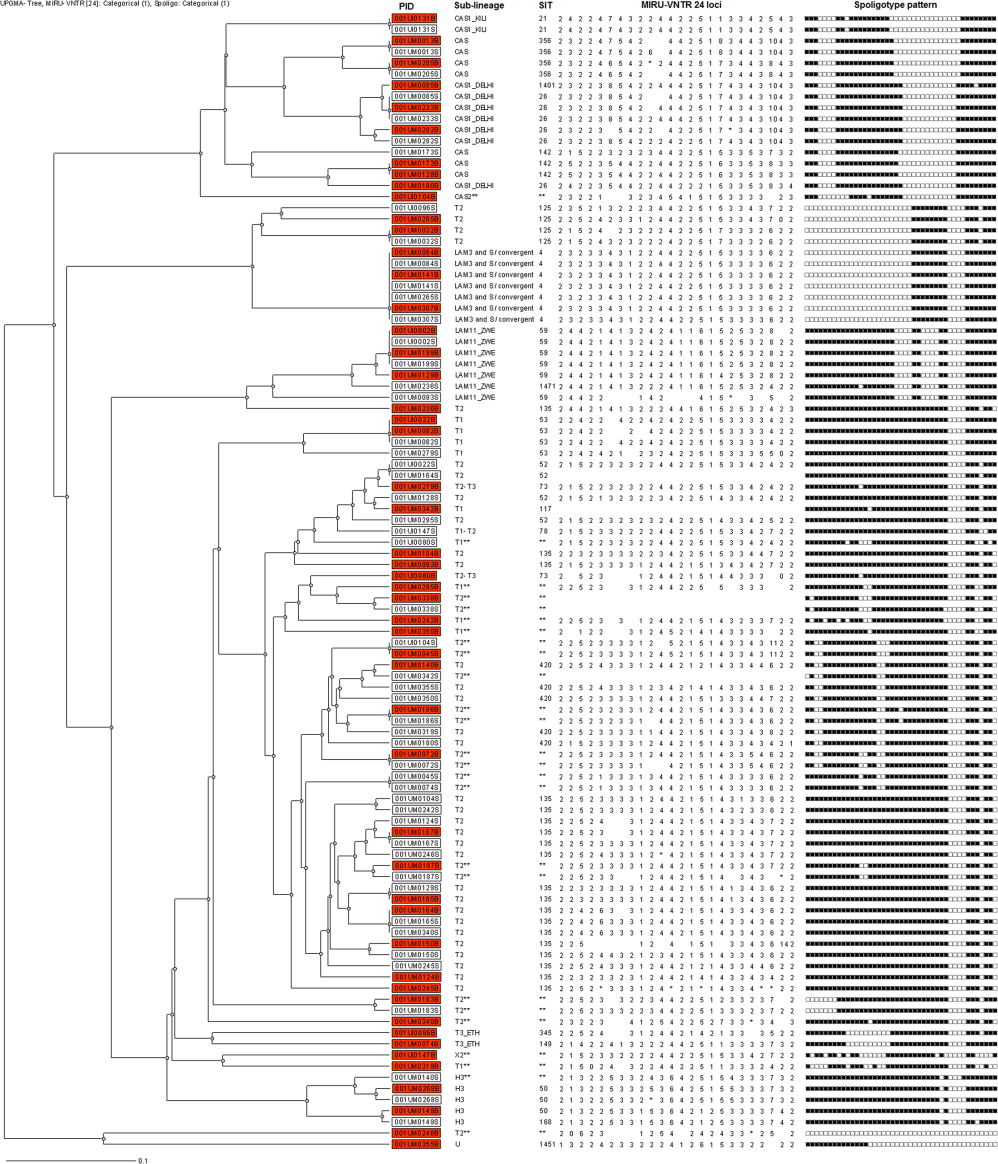
Phylogenetic tree and distribution of *Mycobacterium tuberculosis* lineages by site of disease. SIT = shared international type. H3= Haarlem, CAS= Central-Asian strain, LAM= Latin American and Mediterranean **= according to [29], Completely empty MIRU-VNTR or Spoligotype pattern = not done or uninterpretable, partial empty MIRU-VNTR= non-amplified allele. PID= Patient unique Identification Number, Shaded PID = blood isolates and un-shaded PID= sputum isolates, *=Mixed alleles at a given locus, MIRU-VNTR= Mycobacterial Interspersed Repetitive Unit-Variable-Number Tandem Repeat.

The distribution of *M*. *tuberculosis* lineages by site of disease per participant was as illustrated, Fig 2.

### Diversity of *M*. *tuberculosis* genotypes concurrently isolated from sputum and blood

Of the 51 participants, 25 (49.0%) had concordant and 26 (51.0%) had discordant MTB-genotypes as shown by spoligotyping and/or MIRU-VNTR typing.

Seven patients classified as discordant had isolates with similar spoligotypes, however, they were found to be discordant by the higher resolution 24 loci-MIRU-VNTR genotyping method and hence interpreted as discordant MTB genotypes, S1 Dataset.

Having discordant MTB-genotype was not predicted by MTB-lineage in either blood or sputum, bacillary burden, CD4 cell count or any other clinical characteristic, although it was near-significantly more frequent for the Euro-American lineage in either blood or sputum (p=0.050). Multivariate analysis resulted in statistically unstable models, Table 3.

**Table 3:** Distribution of the global *M*. *tuberculosis* lineages by baseline patient characteristics and *M*. *tuberculosis* –genotypes by concordance of isolates from sputum and blood samples.

Variable	ConcordantN=25; N (%)	DiscordantN=26; N (%)	Odds ratio (95% CI)	p-value
**Euro-American lineage in sputum and/or blood**				
No	6 (24.0)	1 (3.8)	Reference	0.050
Yes	19 (76.0)	25 (96.2)	7.89 (0.99-71.21)	
**East-African-Indian lineage in sputum and/or blood**				
No	19 (76.0)	22 (84.6)	Reference	0.499
Yes	6(24.0)	4 (15.4)	0.58 (0.15-2.22)	
**CD4 count/ mm** ^**3**^				
>50	10 (40.0)	8 (30.8)	Reference	0.565
<50	15 (60.0)	18 (69.2)	1.50 (0.48-4.66)	
**Age group**				
<30	8 (32.0)	11 (42.3)	Reference	0.452
30-39	13 (52.0)	9 (34.6)	0.50 (0.15-1.72)	
=/>40	4 (16.0)	6 (23.1)	1.09 (0.24-4.88)	
**Sex**				
Female	15 (60.0)	10 (38.5)	Reference	0.165
Male	10 (40.0)	16 (61.5)	2.40 (0.79-7.28)	
**Karnofsky performance score**				
70 and above	9 (36.0)	9 (34.6)	Reference	0.918
50-60	16 (64.0)	17 (65.4)	0.94 (0.29-3.00)	
**On ART at enrollment (n=50)**				
Yes	5 (20.8)	5 (19.2)	Reference	0.889
No	19 (79.2)	21 (80.8)	1.11 (0.29-4.18)	
**Previously treated for TB**				
No	22 (88.0)	25 (96.2)	Reference	0.350
Yes	3 (12.0)	1 (3.8)	0.29 (0.03-3.19)	
**Sputum smear status (n=49)**				
Negative	17 (70.8)	14 (56.0)	Reference	0.377
Positive	7 (29.2)	11 (44.0)	1.91 (0.60-6.08)	
**MGIT (sputum) time to detection (days)**				
≥12	9 (36.0)	6 (23.1)	Reference	0.373
7-11	7 (28.0)	12 (46.2)	2.57 (0.60-10.98)	
≤6	9 (36.0)	8 (30.8)	1.33 (0.32-5.58)	
**Blood culture time to detection (days)**				
≥30	8 (32.0)	6 (23.1)	Reference	0.742
22-29	11 (44.0)	12 (46.2)	1.45 (0.37-5.68)	
≤21	6 (24.0)	8 (30.8)	1.78 (0.38-8.29)	
**LJ (sputum) culture colony counts**				
No Growth	5 (20.0)	4 (15.4)	0.565 (0.133-2.399)	0.703
<10	6 (24.0)	4 (15.4)	0.407 (0.115-1.934)	
10-100	12 (48.0)	17 (65.4)	Reference	0.453
>100	2 (8.0)	1 (3.8)	0.353 (0-3.092)	

Abbreviations, LJ- Lowenstein Jensen, MGIT- Mycobacterial Growth Indicator Tube, OR = Odds Ratio.

OR for discordant MTB-lineages, P values for categorical variables were based on the 2-sided Fisher’s exact test.

Two of the fifty-one patients (4%) had MIRU-VNTR patterns showing evidence of mixed infection and both were L4. In one pair, one pattern was found in both blood and sputum, mixed with another pattern in blood. In another pair, the mixed pattern in sputum did not match the pattern in blood, for a total of three genotypes in the same patient.

DST results were available for 87 of 102 samples with viable bacilli from blood and sputum. All isolates tested were susceptible to streptomycin and rifampicin, while one isolate was resistant at 0.2 μg/ml of isoniazid but susceptible at 1.0 μg/ml. Three pairs of isolates (sputum and blood), two from patients with discordant and one with concordant MTB genotypes (T2), showed resistance to ethambutol at 5.0 μg/ml.

## Discussion

In this relatively large study of concurrent sputum and blood isolates we identified a high rate of MTB genotypic discordance. While these HIV-infected patients predominantly had low CD4 cell counts, neither these counts, nor other clinical characteristics, predicted genotypic discordance. Lineages or sub-lineages were not associated with site of disease, but strains of the Euro-American lineage appeared more often in discordant pairs, possibly reflecting a higher potential of strains of this lineage for occurring in polyclonal infections in different compartments. However, this association remained short of significance (p=0.050) and we were unable to adjust for possible confounding. The probability of finding discordant strains within this lineage may have been larger than the probability of finding discordant strains with the East African–Indian lineage simply because of their larger numbers. The high discordance rate documented in this population is not dissimilar to that of much smaller earlier studies of sample sizes up to fourteen pairs [9, 10]. This finding reflects a high prevalence of mixed MTB infections within the same TB patients, potentially even underestimated due to a second, third, etc. infection having been missed, as *in vitro* culture may have allowed one strain to predominate[30]. Indeed, our study found only 4% mixed infection within compartments, similar to the 7% found in a previous study [31]. These multiple MTB strains may have been acquired together (due to exposure to a patient with mixed infection) but are more likely due to exposure to different MTB strains at different times [13, 32]. MTB infection can lead to immediate and/or severe presentation of TB disease, especially in patients with HIV infection [33], or delayed development of TB disease due to *M*. *tuberculosis* persisting in several sites and cell types, which might constitute reservoirs from where infection can reactivate to produce EPTB, with or without lung involvement [34, 35]. Moreover, a recent study showed advanced immune suppression to be associated with increased prevalence of mixed-strain MTB infection[36] which may lead to selection of the most adapted strain to be disseminated. Although not compared with concurrent pulmonary TB, EPTB has recently been found to be genotypically heterogeneous as revealed by whole genome sequence analysis [11]. A recent similar study on *Mycobacterium avium* complex (MAC) found high genetic diversity in strains that cause pulmonary and disseminated disease [37].

As previously described in Uganda [38], we found T2-lineage as the most common lineage in our population. The lack of an association with particular strain lineages suggests that host factors predominate in the breakdown towards mycobacteremia. We presumed that our cohort with weak cellular immunity were more likely to be infected with any lineage irrespective of fitness. Overall in the main study we had high (42.9%) levels of mycobacteremia in patients ≤100 CD4 cells/mm^3^ [21]. There was no statistically significant association of MTB-lineages with CD4 cell count. This could be due to the fact that most of the participants had <50 CD4 cells/mm^3^, which patients are predisposed to severe forms of EPTB [33, 36]. Indeed, other studies have found no association of lineage with site of disease, suggesting that disease presentation is largely determined by host and/or environmental factors [39, 40]. However, such an association between lineages in their affinity for either the pulmonary (sputum) or the extrapulmonary (blood) compartment may become apparent with even larger studies. Novel deep sequencing techniques could help determine the true extent of genetic diversity in each compartment, yet have not been employed here.

As previously documented, we found high concordance of DST results on isolates from sputum and blood [9, 12, 41], and the low rates of drug resistance could be country specific [42].

Limitations of our study include the methods used to conclusively discriminate non-clonal variants. The classification of isolates with one locus difference as concordant could have underestimated the discordance rate, yet is sufficiently conservative to allow for clonal variants to be classified as concordant [24, 26, 43]. Furthermore use of DNA from cultured isolates may have introduced bias for detection of mixed infection [30], however, since we sampled the entire culture for typing, the bias is likely to be less compared to if we had sampled single colonies for typing. Also one would expect patients previously treated for TB to have more genetic discordance, however, only 1 of the 4 previously treated TB patients had discordant MTB genotypes in sputum compared to blood. Moreover, as our findings are mostly from patients with advanced immunosuppression, which group is at highest risk of mycobacteremia, and largely relate to the predominant lineages of the *M*. *tuberculosis complex* in Uganda[38], they may not be generalizable to patients with higher CD4 counts and those infected with other lineages.

Although we document an unexpectedly high discordance rate of MTB genotypes in this population, rigorous quality controls implemented in the GCP-compliant ‘parent’ study decrease the likelihood that this high discordance rate can be attributed to administrative or laboratory related errors.

## Conclusions

Our study reveals high rates of discordance of MTB strains in sputum and blood samples of HIV-infected TB patients, and these findings may reflect underestimation of the true prevalence of mixed MTB infection. In either case, mixed infections of drug-susceptible and drug resistant strains may be easily missed if only one compartment is sampled, such as in sputum testing with Xpert MTB/RIF as is now recommended for PLWHA but mainly optimized for sputum samples[44–46]. Future studies on the course of sequential infections and subsequent reactivations, possibly in experimental animals, are needed to understand what triggers concurrent disease with more than one *M*. *tuberculosis* strain.

## Supporting Information

S1 Dataset Laboratory and clinical information for concurrent *Mycobacterium tuberculosis* isolates from sputum and blood of HIV-infected individuals. (XLS)
